# A Comparison of the Device-Related Complications of Intramedullary Lengthening Nails Using a New Classification System

**DOI:** 10.1155/2017/8032510

**Published:** 2017-10-09

**Authors:** Dong Hoon Lee, Sungmin Kim, Jung Woo Lee, Hoon Park, Tae Yoon Kim, Hyun Woo Kim

**Affiliations:** ^1^Division of Orthopaedic Surgery, Severance Children's Hospital, Yonsei University College of Medicine, 50 Yonsei-ro, Seodaemun-gu, Seoul 03722, Republic of Korea; ^2^Department of Orthopaedic Surgery, Gangnam Severance Hospital, Yonsei University College of Medicine, 211 Eonju-ro, Gangnam-gu, Seoul 06273, Republic of Korea; ^3^Jung-Hwa Girls High School, Chungho-ro 84, Suseong-gu, Daegu 706-819, Republic of Korea

## Abstract

The purpose of this study was to understand the pros and cons of the lengthening nails which have their own mechanical mechanism; we propose a classification for “device-related complications” arising from mechanical properties of the nail itself. From March 2010 to March 2014, 115 segments of lower limb lengthening were performed using intramedullary lengthening nails (35 ISKD, 34 PRECICE1, and 46 PRECICE2). Device-related complications were sorted into three categories according to a new classification: distraction control-related (type I), stability related (type II), and other device-related (type III); these were subdivided using Paley's concept of problems (a), obstacles (b), and sequel (c). Most common complications were distraction mechanism issues (type I) in ISKD and mechanical strength related ones (type II) in PRECICE1 and PRECICE2. Sixty percent (21/35) of ISKD had device-related problems. In PRECICE1 group, 8.8% (3/34) had device-related problems, and 8.8% (3/34) showed device-related obstacle. In PRECICE2, forty-four percent (20/46) had device-related problems. In conclusion, a new classification showed more clearly the differences of mechanical characteristics of different nails. The most essential thing of future lengthening nail development is minimizing the types I and II complications. Further study is necessary to compare the mechanical strength and stability of lengthening nails.

## 1. Introduction

Although external fixators are still the gold standard in the limb lengthening field, surgical techniques such as lengthening over nail or lengthening and then nail have been developed to reduce the period of external fixation [1, 2]. The following four devices are the most clinically known: the Fitbone® (WittensteinIntens, Igersheim, Germany), the Albizzia® (DePuy, Villeurbanne, France), the Intramedullary Skeletal Kinetic Distractor (ISKD®; Orthofix Inc., Lewisville, Texas, USA), and the PRECICE® (Nuvasive, San Diego, CA, USA). Each of the lengthening nails has its own characteristic mechanical mechanisms, which could also bring distinct complications.

The Albizzia is a fully implantable nail that is activated by torsion of 20° along the longitudinal axis of the limb [3–5]. The Fitbone—an electronic motorized lengthening nail which does not need rotational motion to be lengthened—has been reported to have 3–17% of device-related reoperation rate [6, 7]. These two devices mostly have been used in Europe and known to be upgraded after those reports. The ISKD is the first FDA-approved intramedullary lengthening nail. It is activated by the clutch mechanism and expected to provide comfortable lengthening. Various problems with rate control, such as runaway, difficult to distract nail, and nondistracting nail, have been reported [8–12]; this has further led to secondary issues such as delayed union/nonunion [8, 12] and severe pain [9], and was finally discontinued from the market. Accordingly, success with limb lengthening using intramedullary lengthening nails highly depends on precise rate control of distraction, pain resulting from the mechanism of lengthening, and mechanical stability of the device—all of which are caused directly by the device.

Recently, magnetically actuated telescopic nail, the PRECICE, the second FDA-approved device, is being widely used around the world [13–16]. Presumed strengths of the PRECICE are precise control of the distraction and the reduced pain during lengthening.

To fully understand the pros and cons of each device, analyses should divide complications resulting from the device itself from those that are not related to the device. In this study, authors propose a new classification specially for the complications that are from the device itself (device-related complications) and compare outcomes for the two FDA-approved nails, ISKD and PRECICE; device-related complications, non-device-related complications, and the change of alignment and length were compared.

## 2. Materials and Methods

### 2.1. Study Design and Subjects

This study includes ISKD, PRECICE, and PRECICE2. We distinguished PRECICE1 and PRECICE2; PRECICE2 is a revised one of initial version of PRECICE. Henceforward, PRECICE2 has been revised to PRECICE2.1, PRECICE2.2, and currently PRECICE2.3. From March 2010 to March 2012, we used ISKD, the only available lengthening nail in our country, and stopped using ISKD in March 2012 due to rate control issue [9], and switched to PRECICE nail in May 2013 when it was available since then. During this period, 115 segments of bones, 88 femurs in 46 patients, and/or 27 tibias in 14 patients underwent lengthening using one of three intramedullary lengthening nails (35 ISKD, 34 PRECICE1, and 46 PRECICE2). All patients underwent lower limb lengthening for stature lengthening or limb length discrepancy. Detailed patient's demographics were tabulated (Table 1). Stature lengthening was the most common etiology because the lengthening nails have not been covered by national health insurance, so we have limitations in using those for disease or posttraumatic cases. This study was approved by the institutional review board at our institution. No patient was lost during follow-up.

### 2.2. Description of Study

The ratio of femur lengthening to tibia lengthening was 26 : 9 in the ISKDs, 28 : 6 in the PRECICE1, and 34 : 12 in the PRECICE2, with no significant difference. Other demographic data including age, sex ratio, body mass index, smoking history, and final length gain showed no differences between groups, except the follow-up period (Table 1).

All surgical procedures were performed by the senior author (DHL) and were similar to the one described in our previous report [9]. Weight-bearing was limited to 20 kg or less regardless the devices; patients were asked to avoid weight-bearing as much as possible during lengthening. At each follow-up visit, radiologic evaluation using the Picture Archiving and Communication System (PACS, GE Healthcare, Barrington, USA) and clinical evaluation including range of motion of the adjacent joint were done by senior author (DHL).

### 2.3. Variables and Outcome Measures

**Figure 1:**
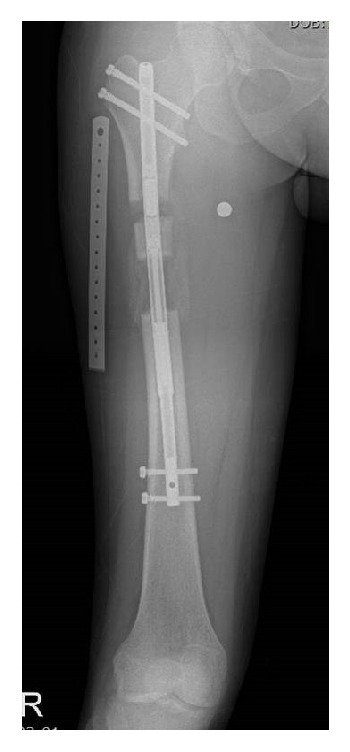
Relatively weak distraction force compared to the thick callus led to the cessation of lengthening. Another osteotomy and further lengthening were done to accomplish the targeted length.

**Figure 2:**
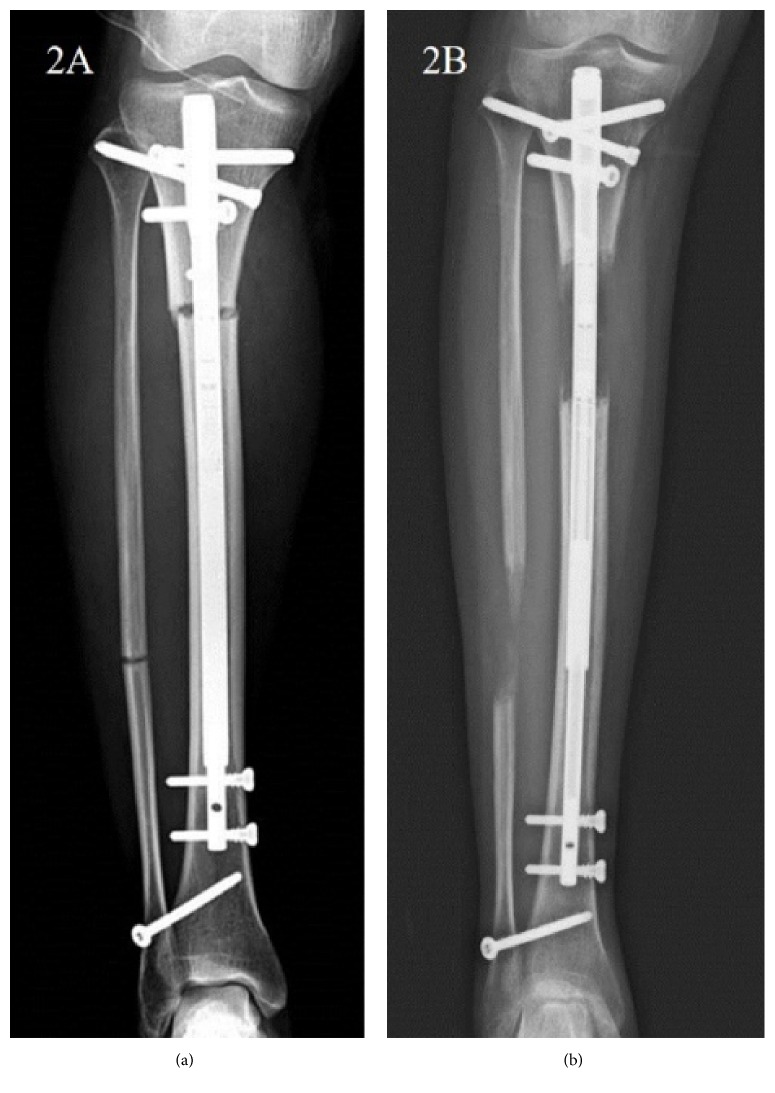
(a) The immediate postoperative plain radiograph showed straight tibia PRECICE nail. (b) The tibia PRECICE nail was bent without breakage after 2 months of lengthening. This was categorized as stability related problem (IIa).

For comparison of device-related complications, they were sorted into three categories: distraction mechanism-related (type I), mechanical strength related (type II), and other device-related (type III). Types I and II are related to the primary function of the nail. Type I is related to the lengthening mechanism and provides the most direct influence on reaching the target length. This includes runaway [9], difficult to distract nail [9], nondistracting nail [9], nonfunctioning nail, and running back [9] (Figure 1). Type II is primarily related to the mechanical strength (stability) of the nail. This can cause instability of the bone, secondary deformity, or prolonged limitation of weight-bearing. Type II complication may include nail bending (Figure 2), nail breakage, or rotational instability. Bending of the nail was defined as over two degrees of the difference between the axis of proximal part and distal part of the nail. Type III is separate from previous two complications and, in principle, does not affect the primary functions of the nail, such as corrosion or adverse reaction of the tissues. Each category was subdivided using Paley's concept of problem (a), obstacle (b), and sequela (c) (in this paper, we changed the term “complication” of Paley's original classification into “sequela” to avoid confusion in terminology) [1, 17] (Table 2).

Non-device-related complications were also collected including joint contracture, deep vein thrombosis, or deep infections. Some complications such as delayed union, nonunion, or temporary hypoesthesia were difficult to clearly classify into device-related complications or non-device-related complications. The rate control (Type I device-related complications) could be thought to affect the incidence of those complications, but we still cannot exclude the possible other host-related factors or surgical technique-related issues as a cause. So, we classified these complications into “non-device-related complications” uniformly in the current study.

The change of alignment and length was evaluated by following measurements; the change of femorotibial angle was checked with orthoradiograph between preoperative and the last follow-up; the change in sagittal plane (femoral bowing in the femur and posterior proximal tibial angle in the tibia) was checked with standing lower extremity-lateral radiograph between preoperative and the last follow-up. The differences between planned and actual length were also checked using orthoradiograph at the last follow-up.

### 2.4. Statistics

The statistical software, R (ver. 2.12 Comprehensive R Archive Network, GNU General Public License, Wien, Austria), was used for all statistical analysis. All continuous variables were tested for normality using the Shapiro-Wilk test and followed a normal distribution. Each continuous measurement is expressed as mean ± SD with range. Chi-squared test of the independence was used for analysis of device-related complication. Because of small sample sizes of non-device-related complications, statistical test could not be conducted for the non-device-related complication with both parametric and nonparametric method. Three of the authors who were orthopedic surgeons measured all radiographic parameters and findings. All of them were blinded to subject information while measuring. We evaluated the intraobserver reliability by repeating all radiologic assessments after 1 week. The intraclass correlation coefficients (ICC) value of the intraobserver reliability was 0.968, and ICC value of the interobserver reliability was 0.913. *P* value of less than 0.05 was considered statistically significant.

## 3. Results

Device-related complications are summarized in Table 3. Overall rate of device-related complication showed 74.3% (26/35), 17.6% (6/34), and 50% (23/46) in ISKD, PRECICE1, and PRECICE2 groups, respectively, which is significantly different.

### 3.1. Type I (Distraction Control-Related Complications)

In the ISKD group, 22 cases (63%) had rate control problems, and four cases (12%) had additional surgery (Ib). In the PRECICE1 group, failure of lengthening during distraction phase due to dense regenerate occurred in two cases (5.9%, Ib) (Figure 1). In the PRECICE2 group, running back occurred in one case (2.2%) which completed lengthening without additional surgery (Ia) and mechanical dysfunction which needed additional surgery (Ib) in one case (2.2%). No cases demonstrated sequelae at the end of the treatment.

### 3.2. Type II (Stability Related Complications)

**Figure 3:**
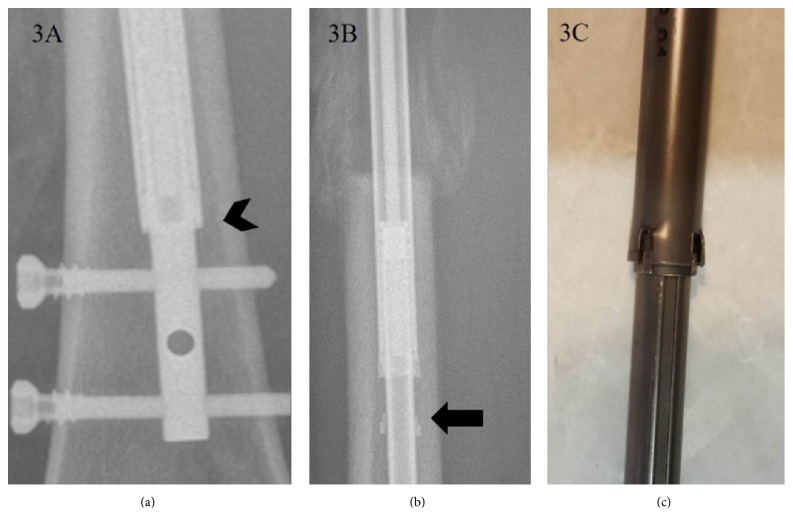
(a) “Rotation coupling” which is at the junction between telescoping rod (arrow head) is intact before lengthening. (b) Disconnection of rotation coupling is seen in PRECICE2 (arrow). Since it was not associated with instability, this was categorized as stability related problem (IIa). (c) Damaged rotation coupling is noticed in the retrieved PRECICE2 nail.

**Figure 4:**
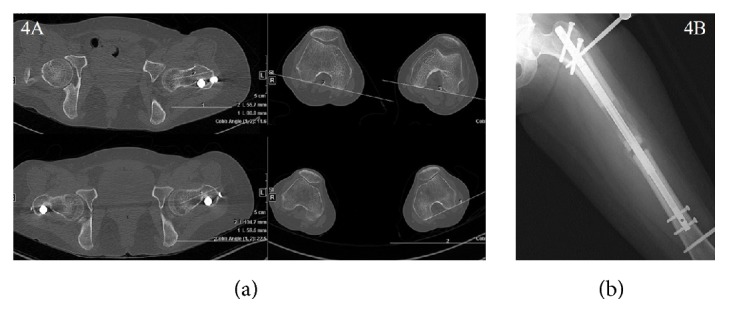
(a) A 38-year-old woman who underwent bilateral femur lengthening complained that she abruptly cannot be able to control her left leg, just after “popping sense” on the leg during early consolidation phase. To confirm the failure of rotational stability of the nail, the CT scans of lower extremity with maximal internal and maximal external rotation were taken, respectively. The femoral rotational alignment changed to about 20 degrees according to the patient's position. (b) Additional mono-fixator was applied to gain a proper rotational alignment and stability until the consolidation is finished.

There were two cases of nail bending without a breakage in the ISKD group. The PRECICE1 group had three bent nails without breakage (IIa) and one nail breakage (2.9%, IIb). In the PRECICE2 group, 7 segments (15.2%) had nail bending without breakage (IIa) (Figure 2). All of this occurred in smallest diameter nail (8.5 mm) which in only available in PRECICE2 and this occupies 35% of 8.5 diameter nail used (7/20) (Table 3). Twelve segments of PRECICE2 (26.1%) showed breakage of rotation coupling without instability (IIa) (Figure 3). All of this occurred in femoral lengthening regardless diameter of the nail. Two segments of PRECICE2 (4.3%; failure of rotational stability) had additional surgery (IIb) (Figure 4).

### 3.3. Type III (Other Device-Related Complications)

No problems were observed for this type of complication.

### 3.4. Device-Related “Problem” (Types Ia, IIa, and IIIa)

Overall rate of device-related problem showed 60% (21/35), 8.8% (3/34), and 43.5% (20/46) in ISKD, PRECICE1, and PRECICE2 group, respectively. Type I occupied 85.7% (18/21) of the problems in ISKD group and type 2 occupied 100% (3/3) and 95% (19/20) in PRECICE 1 and PRECICE2 group, respectively.

### 3.5. Device-Related “Obstacle” (Types Ib, IIb, and IIIb)

Overall rate of device-related obstacle showed 14.3% (5/35), 8.8% (3/34), and 6.5% (3/46) in ISKD, PRECICE1, and PRECICE2 groups, respectively. Type I occupied 80% (4/5) and 66.7% (2/3) of the problems in ISKD and PRECICE 1 groups, respectively. Type 2 occupied 66.7% (2/3) in PRECICE2 group.

### 3.6. Device-Related “Sequela” (Types Ic, IIc, and IIIc)

No cases demonstrated sequelae at the end of the treatment.

### 3.7. Non-Device-Related Complications

In the ISKD group, non-device-related problems, such as transient hypoesthesia, heterotopic ossification, and delayed union, occurred relatively more than the other groups. There were two segments of surgical release due to hip contracture in both ISKD and PRECICE2. There were no cases in all groups with deep infection or nonunion (Table 4).

### 3.8. The Change of Alignment and Length

Acute corrections of the alignment during surgery were done in 8 segments of the tibia to correct 5° to 12° of varus. There was a tendency of valgus deviation in tibial lengthening in all groups. In sagittal plane, the changes were not significant. The differences between planned and actual length were within 3 mm in all cases (Table 5).

## 4. Discussion

Since the 1970s, several intramedullary lengthening nails have been developed to avoid various complications caused by long periods of external fixation [5, 6, 16, 18]. However, many of these have showed some issues associated with the lengthening mechanism or strength of the nail which resulted in nonoptimal results [5, 7–10, 12, 19, 20].

Many issues that occur during lengthening, such as pain, mechanical failure, and imprecise rate control, can be due mostly to the device itself not under control of the surgeon. This differs from external fixation procedures, and it is often difficult to resolve such issues with nonsurgical techniques. Therefore, it is necessary to categorize complications into device-related complications and non-device-related complications.

Thus far, only ISKD and PRECICE have received FDA approval, and each is activated by different mechanisms. In comparing the device-related complications of these nails, this study design has the advantages of being a single-center, single-surgeon series, having no demographic differences among the cohorts. We found that the lowest rate of overall device-related complication was seen in PRECICE1 (17.6%), lowest device-related problem in PRECICE1 (8.8%), and lowest device-related obstacle in PRECICE2 (6.5%). PRECICE nails showed less distraction control-related complications (type I) and less pain, but more mechanical strength related complications (type II) than ISKD.

Several limitations should be mentioned. First, PRECICE2 has been revised up to version 2.3 which tried to upgrade the mechanical strength and stability. The next versions of the product family after PRECICE2 are not included in this study. And the model ISKD is out of production due to its unpredictable rate control. So, the current study does not provide the information of product of the lengthening nails which are available in the market. But authors performed this study to suggest the classification of the device-related complications of lengthening nails. Second, we had a small number of tibia lengthening patients in both groups, which limited our ability to subcategorize or analyze them. A study with a larger sample for tibial lengthening is necessary. Third, this is a consecutive series, with the ISKD being done first, followed by the PRECICE. We cannot exclude the fact that the PRECICE patients did better because the senior author became more experienced with lengthening nails.

The general classifications of complications in limb lengthening were proposed by Caton et al. in 1985 [21], Paley in 1990 [17], Popkov in 1991 [22], Donnan et al. in 2003 [23], and Lascombes et al. in 2012 [24]. Lascombes et al. especially classified the complications in more universal manner after different interventions and differing osteosynthesis methods [24]. However, these classifications are for the general complications of distraction osteogenesis, not specific for the internal bone lengthening devices. Unlike external fixator, lengthening nails have complex mechanical mechanism and this may lead to characteristic complications. Therefore, we believe, apart from the conventional classification system for the general complications of distraction osteogenesis, a new classification for the complications caused by the mechanical problems of the lengthening nail will help in analyzing the character of each nail.

Difficulties of rate control have been reported to be a major disadvantage of ISKD [5, 7, 9, 25]. Predictable control of the distraction rate is critical for callus regeneration, soft tissue adaptation, and pain in distraction osteogenesis. Uncontrolled rate can result in nonunion or premature consolidation, soft tissue damage, and severe pain [5, 7, 9]. Lee et al. observed an abnormal distraction rate of 60% in an ISKD study [9], and many studies have reported poor regeneration linked to ISKD distraction rates that are too high (runaway) [8, 10, 12]. The overall rate of device-related problems (Ia, IIa) was 60% for ISKD, 8.8% for PRECICE1, and 43.5% for PRECICE2. The most common device-related problem of ISKD was type I (distraction control-related), and even though they were managed without an additional surgery, those cases with runaway or difficult to distract nail were hard to manage properly. In PRECICE2, most of device-related problems were nail bending or breakage of rotation coupling without actual instability (type II). Nail bending in PRECICE2 was observed in smallest diameter nail (8.5 mm) which in only available in PRECICE2 and this occupies 35% of 8.5 diameter nail used (7/20). Small diameter nails are necessary for the patients with narrow intramedullary canal, especially in East Asia. However, it seems to be necessary to have a stronger mechanical character in order to avoid the bending phenomenon, since “bending” may change the alignment and be exposed to the risk of nail breakage. Twelve cases using PRECICE2 (26.1%) showed breakage of rotation coupling without instability (IIa). All of this occurred in femoral lengthening regardless diameter of the nail. This shows the importance of higher stability against the rotational force regarding the femoral lengthening nail. The additional surgeries were caused mainly by a nonfunctioning nail and painfully distracting nail (difficult to distract nail) for ISKD (4–33%) [9–11, 19], whereas the main causes were nonfunctioning nail and nail fracture for PRECICE1 (0–19%) [14, 15, 25, 26]. In contrast to the most common device-related obstacle was type I (nondistracting nail or failure of lengthening mechanism) in ISKD; weak distraction force to resist a dense regenerate or nail breakage at the modular portion is the cause of additional surgery in PRECICE1. This has been reported in the previous studies [13, 26] and PRECICE2 is known to be developed to improve these weaknesses. In this study, PRECICE2 showed no more fracture of the nail or weak distraction force. But we observed a new weakness in PRECICE2, failure of rotational stability which resulted in additional surgery.

Pain caused by the lengthening mechanism is especially reported with the use of lengthening nails that require rotating motion; Guichet and Casar reported an average of one additional operation on each limb due to ratcheting under general anesthesia using Albizzia nails [4]. García-Cimbrelo et al. reported 2/24 cases (8%) of ratcheting under general anesthesia for femoral lengthening using Albizzia nails and further reported that, in 3/24 cases (13%), the patients wanted early termination of lengthening due to severe pain during ratcheting [27]. An average VAS for pain of 7-8 has been reported for ISKD lengthening [7], but this study involving the same design reports a lower VAS in PRECICE groups for pain of 3-4 during lengthening motion. We believe that this is due to lack of rotational movement for lengthening and accurate distraction rate control of the PRECICE nail.

Non-implant-related additional surgeries (obstacles) were all caused by hip contracture from femoral lengthening and were successfully released by additional surgery. Delayed unions, all seen in tibial lengthening, were more seen in ISKD group than PRECICE group. Statistical analysis regarding tibial lengthening is not possible due to small number of cases; however, the authors are suggesting that rotational movement gives a bad influence on healing of the tibia which has relatively lower healing potential. A very low rate of non-implant-related complications, especially deep infection and nonunion, in all devices shows promising potential of intramedullary lengthening nails, but we believe this can be achieved only when it is well controlled by an experienced surgeon.

In conclusion, analysis of device-related complications by a new classification showed the differences of mechanical characteristics of different lengthening nails more clearly. The most essential thing in lengthening nails which are to be developed in the future is minimizing the types I and II complications. Further study is necessary to compare the mechanical strength and stability of the upmost revised versions of lengthening nails which are available in the market.

## Figures and Tables

**Table 1:** Descriptive statistics for the patients along nailing machine groups.

Demographic variables	ISKD (*N* = 35)	PRECICE1 *N* = 34	PRECICE2 *N* = 46
Number of patients	19	18	23
Age (years)	28 ± 8	29 ± 7	29 ± 6
Sex (male : female)	20 : 5	15 : 3	17 : 6
Preoperative height (cm)	154 ± 6	161 ± 7	159 ± 6
Body mass index (kg/cm^2^)	22 ± 3	23 ± 3	22 ± 4
Smoking history (yes : no)	9 : 26	5 : 29	7 : 33
Final length gain (mm)	48 ± 8	49 ± 8	51 ± 7
Bones lengthened (femur : tibia)	26 : 9	28 : 6	34 : 12
Duration of follow-up (months)	48 ± 6	18 ± 4	15 ± 5

*Note.* Values are expressed as mean ± standard deviation or as a ratio.

**Table 2:** Device-related complications for internal lengthening devices.

Device-related complications	Description
(I) Distraction control-related	Problem like this can primarily prevent the lengthening target from being met or causes regenerate problems
	Runaway, difficult to distract nail, running back, nondistracting nail
	(a) Problem/(b) obstacle/(c) sequela
(II) Stability related	Problem like this will primarily cause secondary deformity, instability of the bone or limited weight bearing
	Nail bending/breakage, rotational instability
	(a) Problem/(b) obstacle/(c) sequela
(III) Other device-related	Device problems that in principle do not affect the primary functions of the device
	Corrosion, adverse reaction of tissue
	(a) Problem/(b) obstacle/(c) sequela

**Table 3:** Device-related complication.

Complication	ISKD *N* = 35	PRECICE1 *N* = 34	PRECICE2 *N* = 46
Ia	18 (51%)		1 (2.2%)
	Runaway nail: 6 Difficult to distract nail: 12		Running back
Ib	4 (12%)	2 (5.9%)	1 (2.2%)
	Nondistracting nail: 3 Failure of lengthening mechanism: 1	Dense regenerated callus	Nonfunctioning nail
Ic			

Total type I	22 (63%)	2 (5.9%)	2 (5.9%)

IIa	3 (8.6%)	3 (8.8%)	19 (41%)
	Nail bending without breakage: 2	Nail bending without breakage	Nail bending without breakage: 7
	Breakage of rotation coupling without instability: 1		Breakage of rotation coupling without instability: 12
IIb		1 (2.9%)	2 (4.3%)
		Nail breakage	Rotational stability
IIc			

Total type II	3 (8.6%)	4 (11.8%)	21 (46%)

III			

*Note.* Actually, Type III (other device-related complications) complication was not observed.

**Table 4:** Non-implant-related complications.

Complications encountered	ISKD group *N* = 35	PRECICE1 *N* = 34	PRECICE2 *N* = 46	*P* value
*Non-device-related problems*				
Transient hypoesthesia	3 (9%)	1 (2.9%)	1 (2.2%)	0.335
Heterotrophic ossification	3 (9%)	1 (2.9%)	0 (0%)	0.111
Delayed union	4 (11%)	0 (0%)	2 (2.2%)	0.097

*Non-device-related obstacles*	2 (6%)	0 (0%)	2 (2.2%)	0.397

*Non-device-related complications*	0 (0%)	0 (0%)	0 (0%)	

**Table 5:** The change of alignment and length among different lengthening nails.

	ISKD *N* = 35		PRECICE1 *N* = 34		PRECICE2 *N* = 46	
	Femur	Tibia	Femur	Tibia	Femur	Tibia
The change of femorotibial angle (°)^*∗*^	−0.3 (−3 to 3.2)	−1.7 (−3 to 3.2)	−1.1 (−4 to 0.7)	−5.1 (−8.8 to −3.2)	0 (−3.8 to 3.2)	−2.8 (2.1 to −6.7)
The change of sagittal alignment (°)^*∗∗*^	−0.3 (−2 to 1)	0.4 (0 to 1)	−0.5 (−2 to 1)	0 (0 to 0.5)	0 (−2 to 4)	0 (0 to 0.5)
The differences between planned and actual length (mm)	−2 (−4 to 3)	2 (−3 to 3)	−1 (−3 to 2)	1 (−2 to 3)	0 (−2 to 3)	−2 (−2 to 3)

^*∗*^+ means varus and − means valgus alignment; ^*∗∗*^+ means flexion and − means extension alignment.
